# Effects of Baduanjin exercise on working memory in mild cognitive impairment: an fNIRS-based randomized controlled trial

**DOI:** 10.3389/fnagi.2026.1829586

**Published:** 2026-04-23

**Authors:** Can Duan, Yufei Chong, Chanjuan Zheng, Zhengliang Li, Huhu Ai, Yunfei Sun, Pingwei Zhang, Gengyong Zou, Yutong Zhang, Wenguang Xia

**Affiliations:** 1 Department of Rehabilitation, Hubei Provincial Hospital of Integrated Traditional Chinese and Western Medicine, Wuhan, China; 2 Hubei Provincial Clinical Research Center for Stroke Rehabilitation of Integrated Traditional Chinese and Western Medicine, Wuhan, China; 3 Hubei Engineering Research Center of Neuromodulation Technology, Wuhan, China; 4 College of Chinese Medicine, Hubei University of Chinese Medicine, Wuhan, China; 5 Department of Rehabilitation, The Affiliated Hospital of Hubei Provincial Government (Hubei Rehabilitation Hospital), Wuhan, China; 6 Tangjiadun Community Health Service Center, Wuhan, China; 7 Wuhan Red Cross Hospital, Wuhan, China

**Keywords:** Baduanjin, functional near-infrared spectroscopy, mild cognitive impairment, somatosensory cortex, working memory

## Abstract

**Objective:**

This study utilized functional near-infrared spectroscopy (fNIRS) technology to investigate the effects of Baduanjin exercise on activation levels in the dorsolateral prefrontal cortex (DLPFC), frontal eye field (FEF), and somatosensory cortex (SSC) during working memory tasks in patients with mild cognitive impairment (MCI).

**Methods:**

A total of 46 MCI patients were randomly assigned to either a Baduanjin group (*n* = 23) or a control group (*n* = 23). The Baduanjin group underwent a 12-week Baduanjin exercise intervention, while the control group received only health education. Before and after the 12-week intervention, fNIRS was used to measure changes in oxygenated hemoglobin (HbO) levels in 67 channels across the bilateral frontal lobes, temporal lobes, parietal lobes during 1-back tasks. Cognitive function was assessed using the Montreal Cognitive Assessment (MoCA) and the Mini-Mental State Examination (MMSE). Attention, executive function, and visual search ability were evaluated using the Shape Trail Test A and B (STT-A and STT-B). Psychological status was assessed with the Symptom Checklist-90 (SCL-90), and sleep quality was measured using the Pittsburgh Sleep Quality Index (PSQI).

**Results:**

Compared to the control group, the Baduanjin group showed significant improvements in MoCA and MMSE scores after 12 weeks of training, along with significant reductions in STT-B completion time, SCL-90 scores, and PSQI scores. Our fNIRS findings demonstrated a significantly greater recruitment of the right somatosensory cortex (SSC-R) in the Baduanjin group at week 12, which coupled positively with improvements in MoCA scores. Notably, such between-group divergence was absent in the DLPFC and FEF, where activation levels remained comparable between the two groups.

**Conclusion:**

These findings suggest that Baduanjin training can improve cognitive function in MCI patients, particularly in executive function, which may be associated with increased activation in the SSC-R.

## 1 Introduction

As the global demographic shifts toward an aging population, cognitive disorders including dementia and mild cognitive impairment (MCI) have become significant health challenges worldwide. MCI is recognized as a clinical condition that lies between normal cognitive aging and dementia (Bai et al., 2022). Epidemiological investigations have reported that individuals with MCI advance to dementia at an annual rate of approximately 10–15%, representing a 3–10-fold higher progression compared to cognitively healthy older adults (Jia et al., 2020). Most dementia cases are clinically diagnosed during the middle to advanced stages, at which point available therapeutic interventions can only provide transient symptomatic relief, rather than prevent disease onset or reverse its progression. By contrast, MCI is generally considered a potentially reversible condition (Sanz-Blasco et al., 2022). Timely identification and intervention in MCI are deemed essential for dementia prevention, as such measures may delay the onset or progression of Alzheimer’s disease, substantially lower dementia prevalence and healthcare expenditures, and represent the globally acknowledged optimal window for implementing preventive strategies against dementia (Livingston et al., 2020).

Recent investigations have indicated that the initial clinical manifestations of dementia may not primarily entail memory deficits identifiable by conventional verbal memory assessments, but rather involve impairments in working memory, attention, executive function, semantic knowledge, and processing speed. These cognitive dimensions may decline in parallel with, or even precede, the deterioration of verbal memory (Kim et al., 2021). Consequently, working memory dysfunction may represent one of the earliest detectable indicators of neurodegenerative conditions such as MCI and dementia. Working memory constitutes a core cognitive function responsible for the transient storage and manipulation of information. It is intrinsically linked to memory retention, attentional regulation, and executive processes, serving an essential role in a wide range of daily cognitive tasks, including learning, reasoning, decision-making, and communication (Kirova et al., 2015). Preliminary evidence from this study revealed that individuals with MCI exhibit diminished neural activation in the dorsolateral prefrontal cortex (DLPFC), frontal eye field (FEF), and somatosensory cortex (SSC) during 1-back tasks. This reduced task-related cortical activity demonstrated a significant positive association with Montreal Cognitive Assessment (MoCA) scores and appeared to be less susceptible to psychological and sleep-related influences (Duan et al., 2025). These observations highlight the essential involvement of working memory in tracking MCI progression.

Emerging evidence indicates that aerobic exercise, cognitive engagement, and participation in social activities may contribute to the attenuation of further cognitive deterioration (Langa and Levine, 2014). A growing body of research has recently confirmed that physical activity can enhance memory performance in individuals with MCI (Law et al., 2020; Chen et al., 2023; Veronese et al., 2023). Baduanjin, a traditional Chinese mind-body aerobic practice grounded in Traditional Chinese Medicine, is distinguished by its gentle movements, simplicity, and favorable safety profile. In recent years, Baduanjin has been increasingly incorporated into interventions for patients with MCI, yielding measurable improvements in dementia prevention, quality of life, and cognitive function (Lin et al., 2022; Yu et al., 2021). One investigation explicitly reported that Baduanjin training led to improvements in global cognition, executive function, and memory in MCI patients, while also decreasing alertness reaction time and enhancing attentional performance (Xia et al., 2023). Additional studies have suggested that Baduanjin may specifically augment attentional processes such as sustained focus and concentration (Xia et al., 2019). Collectively, these observations imply that Baduanjin exerts beneficial effects on cognitive function in MCI, but the specific neurocognitive mechanisms underlying these outcomes have yet to be fully clarified.

Functional near-infrared spectroscopy (fNIRS) is a non-invasive neuroimaging modality distinguished by its portability, non-invasiveness, ease of use, brief testing duration, cost-effectiveness, resistance to motion artifacts, and relatively high spatial and temporal resolution. This technique facilitates the monitoring and quantification of cerebral hemodynamic responses, which serve as indirect indicators of neural activity via neurovascular coupling mechanisms (Yeung and Chan, 2020). Consequently, fNIRS has been increasingly utilized in research concerning MCI (Lee et al., 2024; Park, 2023). To examine the effects and potential mechanisms of a 12-week Baduanjin training regimen on working memory in individuals with MCI, a prospective randomized controlled trial was designed. The principal aim of this investigation is to evaluate the influence of Baduanjin practice on working memory performance in MCI patients. Furthermore, fNIRS will be employed to investigate how this intervention modulates task-related cortical activity within specific brain regions. It is hypothesized that, following the 12-week intervention, participants in the Baduanjin group will exhibit markedly elevated oxyhemoglobin concentrations in the DLPFC, FEF, and SSC during 1-back task execution, in comparison to the control group.

## 2 Material and methods

### 2.1 Study design

**FIGURE 1:**
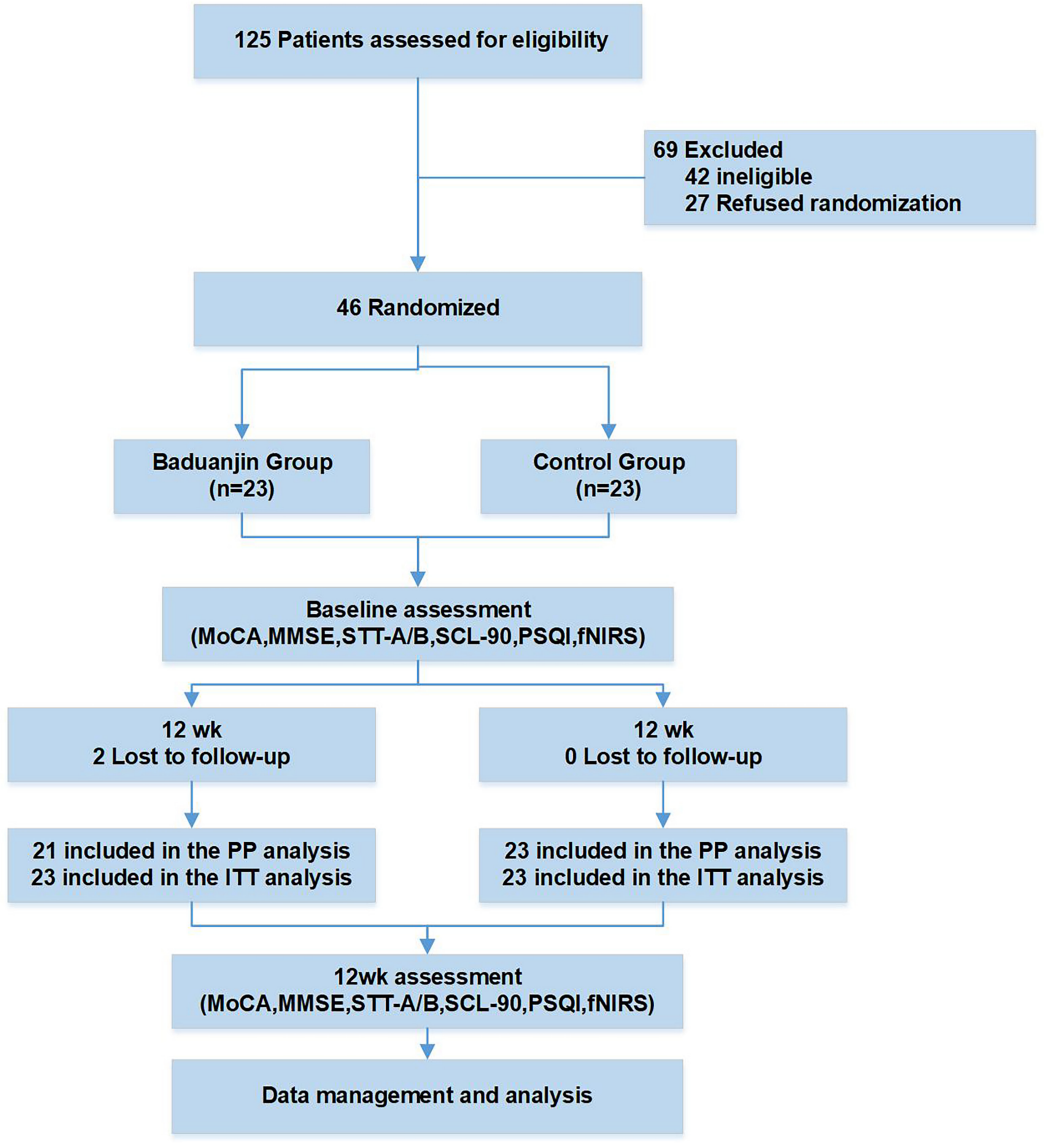
The flowchart of trial procedures.

This study employed a RCT design incorporating allocation concealment and assessor blinding. Participants were randomly assigned to either the Baduanjin group or the control group using a computer-generated random sequence. Random numbers were produced with SPSS 25.0 software. Participants with odd random numbers were assigned to the Baduanjin group, and those with even random numbers were assigned to the control group. Allocation concealment was implemented using sealed opaque envelopes to prevent selection bias. The random allocation sequence was enclosed in sealed, opaque envelopes, which were opened only after eligibility confirmation and baseline assessment to determine group assignment. Forty-six eligible participants were randomly assigned to either a 12-week Baduanjin exercise intervention (comprising 60-min sessions conducted three times per week) or a control condition involving the continuation of routine daily activities over the same period. Qualified evaluators performed extensive assessments using multiple instruments: cognitive performance was evaluated through MoCA and Mini-Mental State Examination (MMSE); executive capabilities, attentiveness, and cognitive processing were measured via Shape Trail Test A and B (STT-A, STT-B); psychological well-being was assessed through the Symptom Checklist-90 (SCL-90); and sleep patterns were examined utilizing the Pittsburgh Sleep Quality Index (PSQI). fNIRS was utilized to monitor bilateral frontal, temporal, and parietal cortex activation during 1-back task execution. All outcome measures were obtained at baseline (pre-intervention) and immediately following completion of the 12-week intervention. The study flowchart and assessment schedule are presented in Figure 1, respectively.

The experimental procedures were executed per the most recent guidelines of the Declaration of Helsinki. Ethical approval was obtained from the local Institutional Review Board, and the study was registered in the International Traditional Medicine Clinical Trials Registry (no. ITMCTR2025000335), and all participants provided written informed consent.

### 2.2 Participants

The sample size was computed utilizing G Power 3.1.9.7 (Faul et al., 2009). An independent samples *t*-test was employed. The MoCA scale was utilized as the primary outcome metric for the final evaluation. Drawing from preliminary pilot findings, a substantial effect size (*d* = 0.966) was computed for sample size estimation. Given α = 0.05 (two-tailed), power = 0.80, and a balanced allocation ratio (1:1), the analysis revealed that 18 participants per group (total *N* = 36) were necessary. To compensate for an anticipated 20% dropout rate, a total of 46 participants (23 per group) were enrolled.

### 2.3 Inclusion criteria

Forty-six participants diagnosed with MCI were recruited from the Tangjiadun Community Health Service Center in Jianghan District, Hubei Province, based on the following inclusion criteria: (1) meeting the diagnostic criteria for MCI in accordance with Petersen’s standards (Petersen, 2004); (2) aged ≥ 60 years, without any history of brain or neurological trauma and in generally stable health; (3) MoCA score ≤ 26; (4) a minimum of 6 years of formal education; (5) right-handed; and (6) no engagement in regular physical activity (defined as structured exercise occurring ≥ 3 times per week for ≥ 20 min per session) within the previous 6 months. Eligible individuals were randomly allocated to either the Baduanjin intervention group or the control group.

### 2.4 Exclusion criteria

Participants were excluded if any of the following conditions were met: (1) uncontrolled hypertension, defined as a systolic blood pressure > 160 mmHg or a diastolic blood pressure > 100 mmHg despite pharmacological intervention; (2) a history of psychiatric disorders (e.g., dissociative disorder, schizophrenia, major depressive disorder) or other primary neurodegenerative dementias; (3) severe dysfunction of the hepatic, renal, hematologic, or endocrine systems; (4) pronounced aphasia, visual impairment (including visual field deficits or spatial neglect), hearing loss, or other impairments that interfered with the completion of assessments; (5) musculoskeletal conditions (e.g., osteoarthritis, joint replacement, fractures) restricting physical activity; (6) cognitive impairment attributed to medications, toxins, or other exogenous agents; (7) concurrent enrollment in other clinical trials with potential to confound the study outcomes.

### 2.5 Intervention protocol

Participants in the Baduanjin group underwent a 12-week intervention comprising the eight standardized Baduanjin exercises, implemented in accordance with the official “Health Qigong: Baduanjin” guidelines issued by the General Administration of Sport of China in 2003. The protocol encompassed ten movements: a preparatory posture, “Holding the Sky with Two Hands to Regulate the Triple Burner,” “Drawing the Bow to Shoot the Eagle,” “Separating Heaven and Earth to Regulate the Spleen and Stomach,” “Looking Backward to Prevent Sickness and Strain,” “Swaying the Head and Shaking the Tail to Relieve Heart Fire,” “Touching the Toes to Strengthen the Kidneys and Waist,” “Clenching the Fists with Angry Eyes to Enhance Strength,” “Bouncing on the Toes to Cure All Diseases,” and a closing posture. During each session, an instructional video was presented via television while live instruction was delivered by a certified Baduanjin instructor with over 5 years of experience. A licensed physician provided oversight throughout the sessions to ensure participant safety. Vital signs, including blood pressure, heart rate, and oxygen saturation, were measured prior to exercise, at 30 min into the session, and following 60 min of exercise to maintain moderate-intensity effort at 50–70% of maximum heart rate (calculated as 220 minus age). The Baduanjin sessions were conducted three times weekly for 12 weeks, with each 60-min session consisting of 10 min of warm-up, 40 min of practice, and 10 min of cool-down. All sessions were documented in training logs. Participants were instructed to abstain from any other forms of physical activity throughout the intervention period.

Participants in the control group were instructed to maintain their usual daily activities without engaging in any additional physical exercise.

### 2.6 fNIRS measurement

**FIGURE 2:**
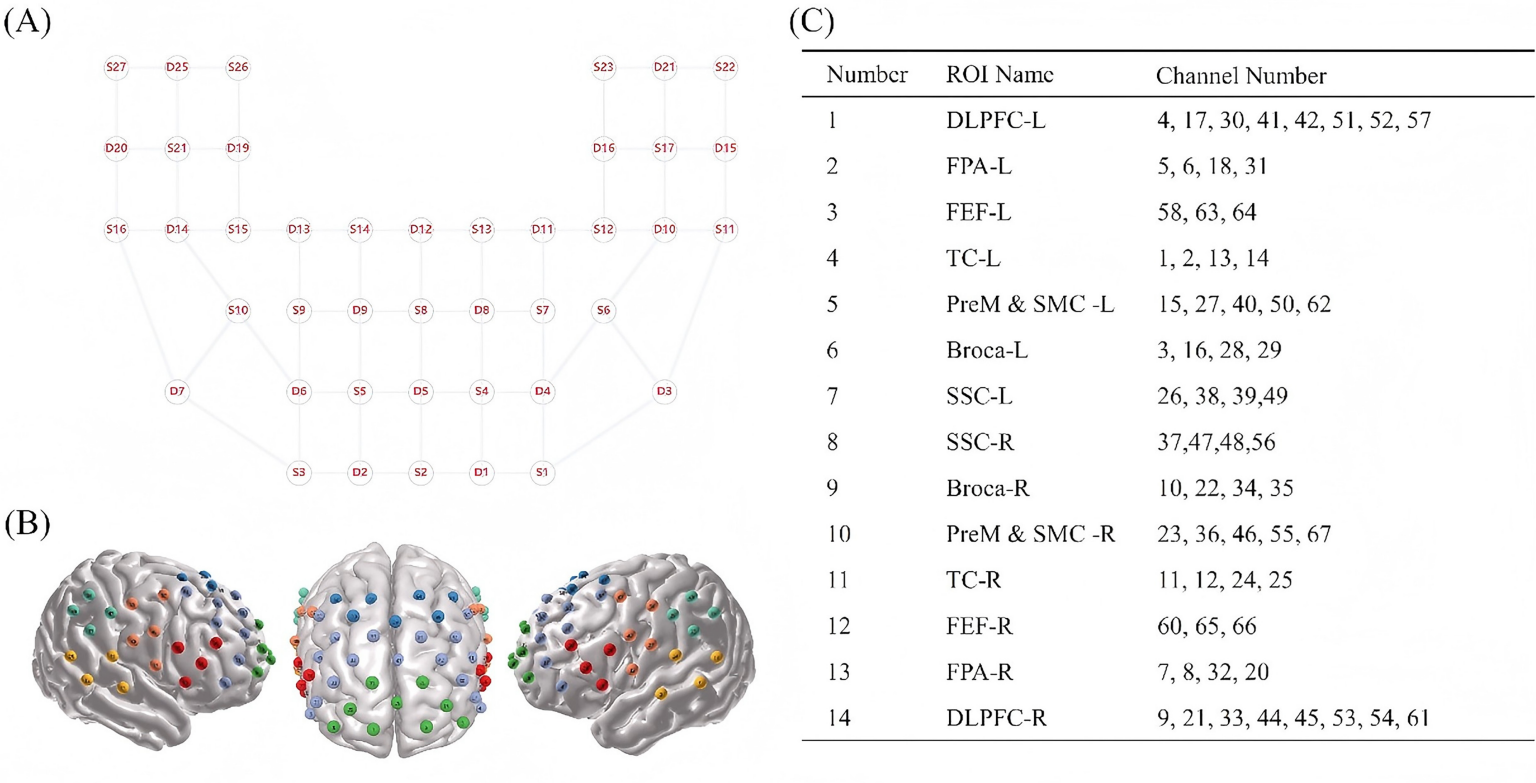
fNIRS 67 channels and 14 ROIs. **(A)** 2D layout of optodes. **(B)** 3D spatial registration of 67 fNIRS channels. **(C)** Assignment of 67 Channels to 14 ROIs, corresponding to 7 anatomically symmetrical brain areas: dorsolateral prefrontal cortex (DLPFC), frontal pole area (FPA), frontal eye field (FEF), temporal cortex (TC), Premotor and Supplementary Motor Cortex (PreM and SMC), Somatosensory Cortex (SSC) and Broca’s area.

The fNIRS data were acquired using the BS-7000 system (Wuhan Znion Technology Co., Ltd., Wuhan, China), which utilizes dual-wavelength laser diodes (690 and 830 nm) and operates at a sampling frequency of 20 Hz. This system consists of 22 sources and 20 detectors, collectively forming 67 channels, with each channel defined by a source-detector pair separated by a probe distance of 3 cm. All probe placements conformed to the international 10–20 system, with probe S2 corresponding to the Fpz point. Spatial coordinates of reference landmarks (Nz, Cz, AL, RL) and optodes were captured using a 3D digitizer (NirMap, Wuhan Znion Technology Co., Ltd., Wuhan, China). Channel registration was precisely projected onto the cortical surface and mapped to Brodmann areas via the NIRS-SPM method (Ye et al., 2009), and subsequently categorized into fourteen ROIs (Figure 2).

### 2.7 1 -back task process

This study employed a digital 1-back task to assess working memory and executive function, requiring participants to determine whether the currently presented digit matched the preceding one, while simultaneously recording fNIRS data to analyze bilateral frontal, temporal, and parietal cortex activation patterns.

**FIGURE 3:**
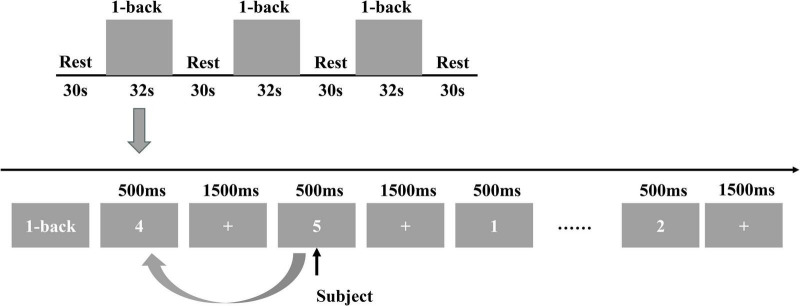
1-back task process.

The entire task comprised the following three phases: (1) Preparation phase (30 s): Included a pre-scan period (0–10 s) and a baseline period (20–30 s). Participants were instructed to relax and fixate on a crosshair displayed on the screen to standardize pre-task brain activity. The fNIRS device synchronously recorded baseline brain activity during this phase. (2) Task phase (156 s): Consisted of three 32-s subtasks. Each subtask began with a 2-s cue, followed by a 500-ms digit presentation. After each digit disappeared, a 1,500-ms crosshair was displayed. Digits were presented 15 times per subtask, requiring participants to judge whether each stimulus matched the preceding digit. The task was repeated three times consecutively, with the fNIRS device synchronously recording brain activity throughout. (3) Recovery phase (30 s): Upon task completion, participants fixated on the crosshair to relax, facilitating a smooth transition from the task state to a resting state. The fNIRS device continued recording brain activity during this phase. The detailed experimental procedure is illustrated in Figure 3.

### 2.8 fNIRS data analysis

fNIRS data analysis was executed utilizing NirMaster software, a specialized platform developed by the device manufacturer specifically for processing fNIRS data. This software provides comprehensive functionalities for data preprocessing, extraction of individual-level features, and statistical analysis and visualization at the group level.

In fNIRS research, the coefficient of variation (CV) is commonly employed as an indicator for evaluating signal quality (Piper et al., 2014; Klein, 2024). Before data preprocessing, CV values were computed for all channels. Channels exhibiting CV values exceeding 15% were classified as low-quality signals (Piper et al., 2014). Participants for whom more than 20% of channels were identified as low-quality were excluded from subsequent analyses. Therefore, two participants were excluded from this study.

The preprocessing pipeline was initiated by converting raw light intensity data into optical density (Delpy et al., 1988). Motion artifacts were addressed through spline interpolation to attenuate transient disturbances (Scholkmann et al., 2010). A band-pass filter ranging from 0.01 to 0.1 Hz was subsequently applied to eliminate physiological noise and baseline drift (Luo and Puthusserypady, 2007; Prince et al., 2003), followed by linear detrending. Hemoglobin concentration changes (oxy-Hb, deoxy-Hb, total-Hb) were then derived using the modified Beer–Lambert law (Kocsis et al., 2006). Oxy-Hb was selected as the primary indicator owing to its superior signal-to-noise ratio and stronger association with cerebral blood flow in comparison to deoxy-Hb (Malonek et al., 1997; Strangman et al., 2002).

To enhance the stability of the concentration response, a 60-s segment was extracted in alignment with each task marker, comprising 10 s of pre-task, 30 s of the task, and 20 s of post-task data. Baseline correction was performed by subtracting the mean value of the pre-task period from each segment. Subsequently, the block-averaged concentration was derived by averaging these corrected segments. A 5-s sliding average was applied to the oxy-Hb signal to mitigate transient motion artifacts and physiological noise. And this 60-s segment was used for the extraction and analysis of task-related hemodynamic responses.

In this study, brain activation elicited by the 1-back task was examined through the application of the general linear model (GLM) (Kiebel et al., 1999; Hou et al., 2021). Within the GLM framework, the observed hemodynamic signal was regarded as the dependent variable and was expressed as a linear combination of experimental conditions or condition contrasts, nuisance covariates, and a residual error term. The resulting beta estimates represented the amplitude of task-evoked activation and served as inputs for subsequent group-level statistical analyses. The GLM analysis in this study was performed based on the 1-back task design, and the time window setting corresponding to this task paradigm was actually adopted.

Previous research has demonstrated that NIRS signals exhibit greater reliability when assessed at the region of interest (ROI) level, as single-channel data are more susceptible to noise, motion-related artifacts, and inter-individual anatomical variability. To improve signal robustness, ROI-level analyses were implemented by averaging the concentration values across all channels contained within each ROI. These mean values were subsequently utilized to compute β-values, which characterized the activation properties of each ROI.

Based on previous studies indicating decreased activation in the DLPFC, FEF, and SSC in patients with MCI during the 1-back task, the present study pre-specified three primary regions of interest (ROIs): DLPFC, FEF, and SSC. The right somatosensory cortex (SSC-R) was included as one of the pre-specified ROIs. fNIRS data analyses were focused on these three *a priori* ROIs to test the pre-specified research hypothesis. Therefore, no additional multiple-comparison correction was performed for the fNIRS analyses.

### 2.9 Behavioral measures

The primary outcome was the MoCA score assessed after 12 weeks of intervention. The MoCA serves as an instrument for evaluating global cognition, producing scores ranging from 0 to 30, with higher scores reflecting superior cognitive performance. Secondary outcomes were determined at the 12-week mark and comprised MMSE, STT-A, STT-B, SCL-90, PSQI, and fNIRS metrics.

### 2.10 Statistical analysis

Statistical analyses were executed utilizing SPSS 25.0 software. Initially, tests of normality were conducted on all datasets. Chi-square tests were employed to examine intergroup differences in sex, hypertension, diabetes, heart disease, and hyperlipidemia. Among the baseline characteristics, age and STT-A scores conformed to normal distributions and were analyzed using independent samples *t*-tests. In contrast, variables violating normality assumptions, such as MoCA scores, MMSE scores, education level, STT-B, PSQI, and SCL-90, were subjected to Mann-Whitney U tests. The significance level (α) was established at 0.05 for all statistical procedures, with P ≤ 0.05 regarded as statistically significant and *P* ≤ 0.01 interpreted as highly significant. Categorical data were reported as frequencies, percentages, or composition ratios, while continuous variables were presented as mean ± standard deviation or median (interquartile range).

Intention-to-treat (ITT) analysis was performed including all randomized participants (*n* = 46), regardless of whether they completed the 12-week intervention. Per-protocol (PP) analysis was also conducted for participants who completed the entire intervention program without major protocol violations (*n* = 44). For the 2 participants who dropped out during the intervention, final post-intervention outcome data could not be collected. Therefore, the last observation carried forward (LOCF) method was used for missing data imputation, and their baseline data collected at enrollment were used as the post-intervention data for subsequent statistical analysis, to ensure that all randomized participants were included in the ITT analysis and to minimize the impact of missing data on the study results.

For fNIRS data collected at two treatment time points (baseline and 12 weeks), repeated measures ANOVA was employed to assess temporal variations. Furthermore, a 2 × 2 mixed ANOVA was conducted to compare fNIRS measurements between the experimental and control groups at baseline and after 12 weeks. In this model, group (experimental vs. control) was treated as a between-subject factor, while time point (baseline vs. 12 weeks) was treated as a within-subject factor. The sphericity assumption in ANOVA was evaluated using Mauchly’s test. When violations were identified, the Greenhouse-Geisser correction was applied to improve result robustness. A significance level of 0.05 was adopted.

## 3 Results

### 3.1 Participant characteristics

A total of 46 participants were randomly allocated to either the treatment group (*n* = 23) or the control group (*n* = 23), and an ITT analysis was conducted. Among the 186 individuals screened, 46 were enrolled and subsequently randomized. Of these, 44 participants completed the 12-week evaluations, and all individuals were included in both ITT and PP analyses. Baseline demographic and clinical characteristics of the participants are presented in Table 1.

**TABLE 1:** Demographic and clinical characteristics of participants (ITT Analysis).

Participants characteristics	Treat group		Control group		Test statistics
	*N*	Mean ± SD	*N*	Mean ± SD	
Sex (male/female)	23 (5/18)		23 (5/18)		χ2 = 0.000,*P* = 1
Hypertension (yes/no)	23 (11/12)		23 (12/11)		χ2 = 0.000,*P* = 1
Diabetes mellitus (yes/no)	23 (4/19)		23(8/15)		χ2 = 1.015,*P* = 0.314
Cardiac disease (yes/no)	23(2/21)		23(3/20)		χ2 = 0.000,*P* = 1
Hyperlipidemia (yes/no)	23(4/19)		23(6/17)		χ2 = 0.128,*P* = 0.721
Age (years)	23	70.09 ± 3.704	23	72.52 ± 4.660	*t* = −1.962,*P* = 0.056
Education	23	11.00 ± 2.431	23	12.22 ± 2.522	*t* = −1.667,*P* = 0.103
MoCA	23	22.96 ± 1.846	23	22.74 ± 1.421	*t* = 0.448,*P* = 0.657
MMSE	23	26.43 ± 2.428	23	26.39 ± 1.340	*t* = 0.075,*P* = 0.940
STT-A(s)	23	67.04 ± 26.842	23	69.87 ± 19.63	*t* = −0.408,*P* = 0.686
STT-B(s)	23	177.87 ± 44.026	23	204.74 ± 73.838	*t* = −1.499,*P* = 0.141
SCL-90	23	118.78 ± 23.063	23	120.74 ± 22.610	*t* = −0.291,*P* = 0.773
PSQI	23	7.61 ± 3.963	23	7.22 ± 4.056	*t* = 0.331,*P* = 0.742

### 3.2 Primary outcomes

In the ITT analysis at 12 weeks, markedly higher mean MoCA scores were observed in the treatment group compared to the control group (26.65 ± 1.695 vs. 22.30 ± 2.530, *P* < 0.001). Likewise, in the PP analysis, the treatment group exhibited a markedly greater improvement in MoCA scores relative to the control group (26.86 ± 1.621 vs. 22.30 ± 2.530, *P* < 0.001) (Table 2).

**TABLE 2:** Comparison of MoCA scores (ITT and PP analyses).

Measurement time	Study group		Test statistics	Effect size	95% CI
	Baduanjin group	Control group			
ITT analysis MoCA Score, Mean ± SD					
Baseline	22.96 ± 1.846	22.74 ± 1.421	*t* = 0.448, *P* = 0.657	η^2^ = 0.005	(−0.762, 1.196)
12 wk	26.65 ± 1.695	22.30 ± 2.530	*t* = 6.846, P<0.001*	η^2^ = 0.516	(3.068, 5.628)
Test statistics	*t* = −9.132, *P*<0.001*	*t* = −0.859, *P* = 0.400			
Effect size	*d* = 1.904	*d* = 0.179			
95% CI	(−4.535, −2.856)	(−0.615, 1.485)			
PP analysis MoCA Score, Mean ± SD					
Baseline	22.81 ± 1.861	22.74 ± 1.421	*t* = 0.142, *P* = 0.888	η^2^ = 0.000	(−0.932, 1.072)
12 wk	26.86 ± 1.621	22.30 ± 2.530	*t* = 7.029, *P*<0.001*	η^2^ = 0.540	(3.246, 5.860)
Test statistics	*t* = −11.399, *P*<0.001*	*t* = −0.859, *P* = 0.400			
Effect size	*d* = 2.488	*d* = 0.179			
95% CI	(−4.788, −3.307)	(−0.615, 1.485)			

*Indicates *P*-value is statistically significant.

### 3.3 Secondary outcomes

In the ITT analysis at 12 weeks, markedly higher MMSE scores were recorded in the treatment group compared to the control group (28.39 ± 1.406 vs. 26.43 ± 1.674, *P* < 0.001). Concurrently, the treatment group yielded markedly lower scores than the control group on STT-A (59.00 ± 20.068 vs. 71.57 ± 20.124, *P* = 0.040), STT-B (154.22 ± 41.366 vs. 197.91 ± 61.025, *P* = 0.004), SCL-90 (97.78 ± 8.235 vs. 119.78 ± 18.882, *P*<0.001), and PSQI (4.91 ± 2.295 vs. 7.83 ± 4.053, *P* = 0.004).

Similarly, in the PP analysis, markedly improved MMSE scores were observed in the treatment group relative to the control group (28.52 ± 1.365 vs. 26.43 ± 1.674, *P* < 0.001), accompanied by markedly lower scores in STT-B (150.48 ± 40.930 vs. 197.91 ± 61.025, *P* = 0.0045), SCL-90 (97.38 ± 8.500 vs. 119.78 ± 18.882, *P* < 0.001), and PSQI (4.81 ± 2.294 vs. 7.83 ± 4.053, *P* = 0.005). Although a declining trend in STT-A scores was evident in the treatment group (60.50 ± 20.188 vs. 71.57 ± 20.124, *P* = 0.078), the between-group difference did not reach statistical significance (Table 3).

**TABLE 3:** Comparison of secondary outcomes (ITT and PP analyses).

Measurement time	Study group		Test statistics	Effect size	95% CI
	Baduanjin group	Control group			
ITT Analysis MMSE Score, Mean ± SD					
Baseline	26.43 ± 2.428	26.39 ± 1.340	*t* = 0.075, *P* = 0.940	η^2^ = 0.000	(−1.122, 1.209)
12 wk	28.39 ± 1.406	26.43 ± 1.674	*t* = 4.292, *P*<0.001*	η^2^ = 0.295	(1.038, 2.875)
Test statistics	*t* = −5.083, *P*<0.001*	*t* = −0.176, *P* = 0.862			
Effect size	*d* = 1.060	*d* = 0.036			
95% CI	(−2.755, −1.158)	(−0.556, 0.469)			
PP Analysis MMSE Score, Mean ± SD					
Baseline	26.38 ± 2.519	26.39 ± 1.340	*t* = −0.017, *P* = 0.986	η^2^ = 0.000	(−1.223, 1.202)
12 wk	28.52 ± 1.365	26.43 ± 1.674	*t* = 4.511, *P*<0.001*	η^2^ = 0.326	(1.154, 3.024)
Test statistics	*t* = −5.382, *P*<0.001*	*t* = −0.176, *P* = 0.862			
Effect size	*d* = 1.175	*d* = 0.036			
95% CI	(−2.973, −1.312)	(−0.556, 0.469)			
ITT analysis STT-A Score, Mean ± SD					
Baseline	67.04 ± 26.842	69.87 ± 19.634	*t* = −0.408, *P* = 0.686	η^2^ = 0.004	(−16.801, 11.149)
12 wk	59.00 ± 20.068	71.57 ± 20.124	*t* = 2.120, *P* = 0.040*	η^2^ = 0.093	(−24.508, −0.622)
Test statistics	*t* = 1.490, *P* = 0.150	*t* = −0.306, *P* = 0.762			
Effect size	*d* = 0.311	*d* = 0.064			
95% CI	(−3.153, 19.240)	(−13.173, 9.782)			
PP analysis STT-A Score, Mean ± SD					
Baseline	69.38 ± 26.850	69.87 ± 19.634	*t* = −0.069, *P* = 0.945	η^2^ = 0.000	(−14.711, 13.734)
12 wk	60.5 ± 20.188	71.57 ± 20.124	*t* = −1.807, *P* = 0.078	η^2^ = 0.072	(−23.270, 1.282)
*P*-value	*t* = 1.494, *P* = 0.151	*t* = −0.306, *P* = 0.762			
Effect size	*d* = 0.326	*d* = 0.064			
95% CI	(−3.492, 21.111)	(−13.173, 9.782)			
ITT Analysis STT-B Score, Mean ± SD					
Baseline	177.87 ± 44.026	204.74 ± 73.838	*t* = −1.499, *P* = 0.141	η^2^ = 0.049	(−62.996, 9.257)
12 wk	154.22 ± 41.366	197.91 ± 61.025	*t* = −2.842, *P* = 0.007*	η^2^ = 0.155	(−74.677, −12.715)
Test statistics	*t* = 2.733, *P* = 0.012*	*t* = 0.381, *P* = 0.707			
Effect size	*d* = 0.570	*d* = 0.080			
95% CI	(5.706, 41.598)	(−30.303, 43.955)			
PP analysis STT-B Score, Mean ± SD					
Baseline	176.38 ± 45.468	204.74 ± 73.838	*t* = −1.516, *P* = 0.137	η^2^ = 0.052	(−66.104, 9.388)
12 wk	150.48 ± 40.930	197.91 ± 61.025	*t* = −2.998, *P* = 0.005*	η^2^ = 0.176	(−79.369, −15.504)
*P*-value	*t* = 2.773, *P* = 0.012*	*t* = 0.381, *P* = 0.707			
Effect size	*d* = 0.605	*d* = 0.080			
95% CI	(6.415, 45.394)	(−30.303, 43.955)			
ITT analysis SCL-90 Score, Mean ± SD					
Baseline	118.78 ± 23.063	120.74 ± 22.610	*t* = −0.291, *P* = 0.773	η^2^ = 0.002	(−15.529, 11.616)
12 wk	97.78 ± 8.235	119.78 ± 18.882	*t* = −5.122, *P*<0.001*	η^2^ = 0.374	(−30.657, −13.343)
Test statistics	*t* = 4.118, *P*<0.001*	*t* = 0.511, *P* = 0.615			
Effect size	*d* = 0.859	*d* = 0.194			
95% CI	(5.099, 10.424)	(−2.928, 4.841)			
PP analysis SCL-90 Score, Mean ± SD					
Baseline	120.38 ± 23.534	120.74 ± 22.610	*t* = −0.051, *P* = 0.959	η^2^ = 0.000	(−14.401, 13.674)
12 wk	97.38 ± 8.500	119.78 ± 18.882	*t* = −4.991, *P*<0.001*	η^2^ = 0.176	(−31.460, −13.343)
Test statistics	*t* = 4.269, *P*<0.001*	*t* = 0.511, *P* = 0.615			
Effect size	*d* = 0.932	*d* = 0.194			
95% CI	(11.761, 34.239)	(−2.928, 4.841)			
ITT analysis PSQI Score, Mean ± SD					
Baseline	7.61 ± 3.963	7.22 ± 4.056	*t* = 0.331, *P* = 0.742	η^2^ = 0.002	(−1.992, 2.774)
12 wk	4.91 ± 2.295	7.83 ± 4.053	*t* = −3.000, *P* = 0.004*	η^2^ = 0.170	(−4.870, −0.956)
Test statistics	*t* = 3.484, *P* = 0.002*	*t* = −0.928, *P* = 0.363			
Effect size	*d* = 0.727	*d* = 0.107			
95% CI	(0.774, 1.091)	(−1.968, 0.751)			
PP analysis PSQI Score, Mean ± SD					
Baseline	7.76 ± 4.073	7.22 ± 4.056	*t* = 0.444, *P* = 0.659	η^2^ = 0.005	(−1.931, 3.020)
12 wk	4.81 ± 2.294	7.83 ± 4.053	*t* = −2.999, *P* = 0.005*	η^2^ = 0.372	(−5.047, −0.986)
Test statistics	*t* = 3.572, *P* = 0.002*	*t* = −0.928, *P* = 0.363			
Effect size	*d* = 0.779	*d* = 0.107			
95% CI	(1.228, 4.677)	(−1.968, 0.751)			

*Indicates *P*-value is statistically significant.

### 3.4 Comparison of outcome changes

In the ITT analysis at 12 weeks, markedly greater improvements in MoCA score changes were observed in the treatment group compared to the control group (3.70 ± 1.941 vs. −0.43 ± 2.428, *P* < 0.001), with a negative mean change noted in the control group, indicating a declining trend. Substantially greater enhancements were also identified in MMSE (1.96 ± 1.846 vs. 0.04 ± 1.186, *P* < 0.001), SCL-90 (−21.00 ± 24.456 vs. −0.96 ± 8.982, *P* = 0.001), and PSQI (−2.70 ± 3.710 vs. 0.61 ± 3.144, *P* = 0.002). Although improvements in STT-A (−8.04 ± 25.892 vs. 1.70 ± 26.542, *P* = 0.214) and STT-B (−23.65 ± 41.50 vs. −6.83 ± 85.861, *P* = 0.402) were evident in the treatment group, the between-group differences did not reach statistical significance.

**FIGURE 4:**
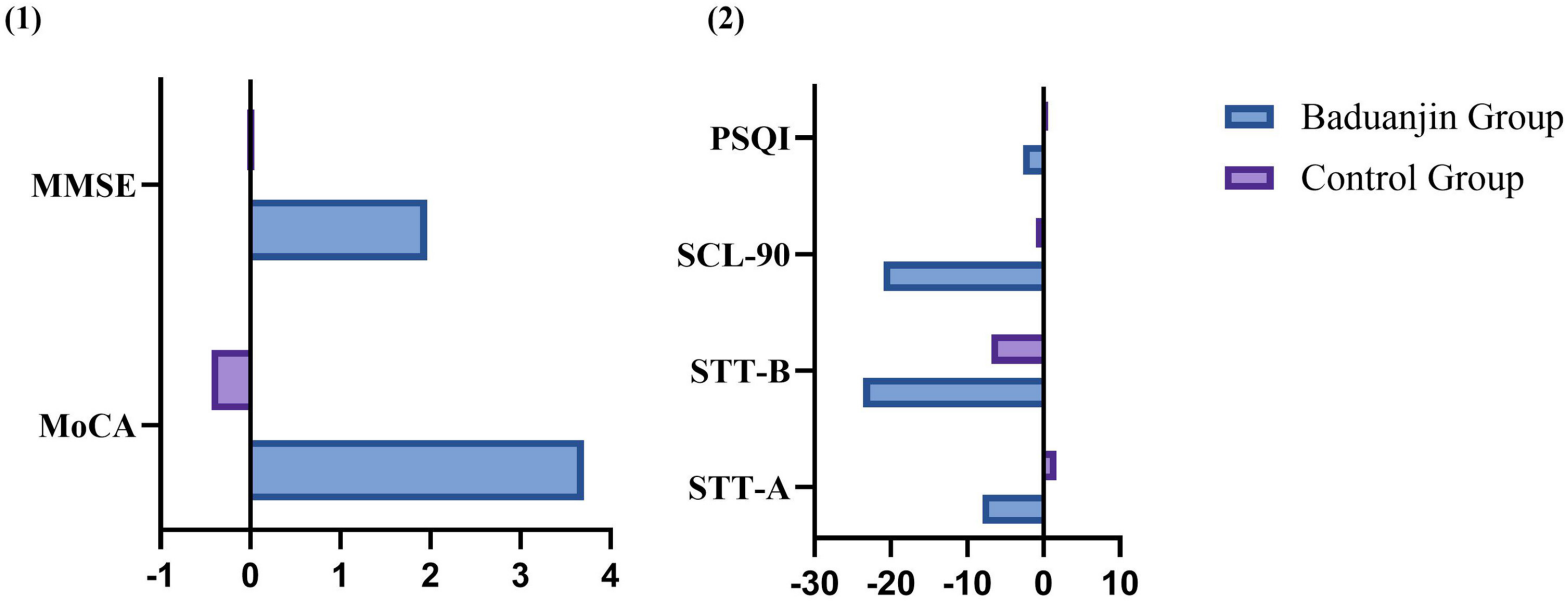
Comparison of changes between the two groups (ITT Analysis). **(1)** MoCA (*P* < 0.01), MMSE (*P* < 0.01); **(2)** STT-A (*P* > 0.05), STT-B (*P* > 0.05), SCL-90 (*P* < 0.01), PSQI (*P* < 0.01).

**FIGURE 5:**
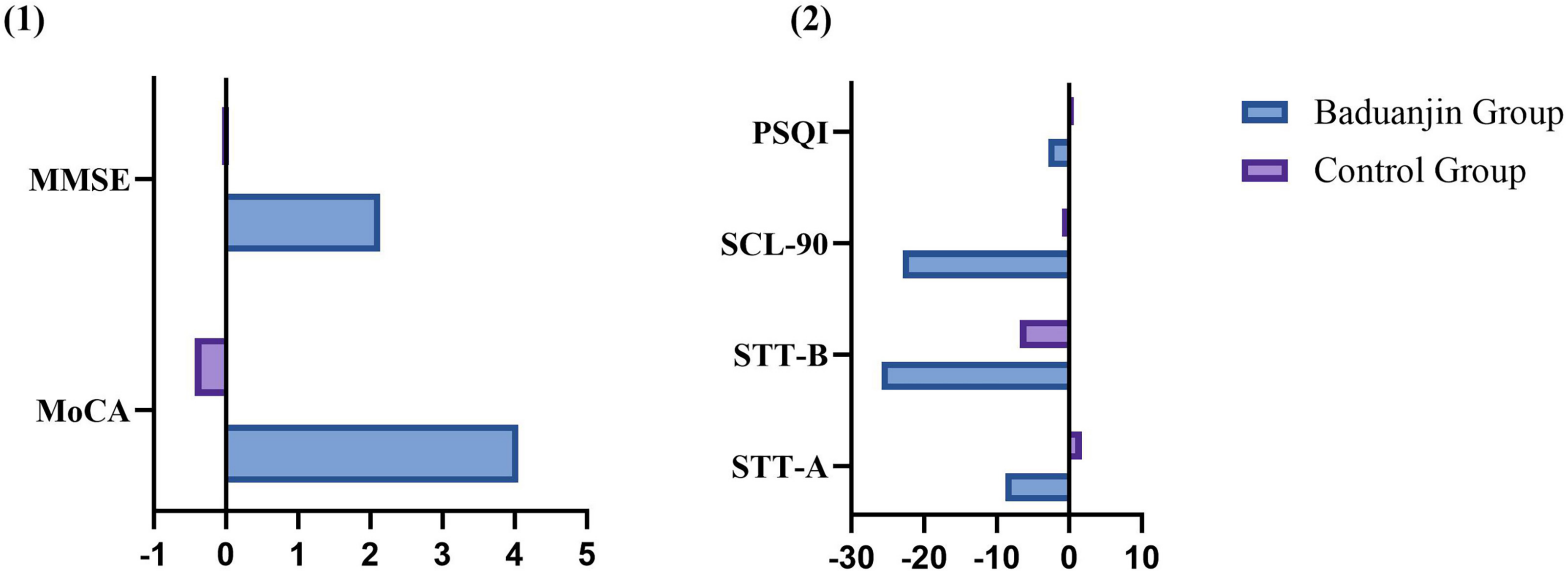
Comparison of changes between the two groups (PP Analysis). **(1)** MoCA (*P* ≤ 0.01), MMSE (*P* ≤ 0.01); **(2)** STT-A (*P* ≥ 0.05), STT-B (*P* ≥ 0.05), SCL-90 (*P* ≤ 0.01), PSQI (*P* ≤ 0.01).

In the PP analysis at 12 weeks, the treatment group exhibited markedly greater improvements in MoCA score changes (4.05 ± 1.627 vs. −0.43 ± 2.428, *P* < 0.001), MMSE (2.14 ± 1.824 vs. 0.04 ± 1.186, *P*<0.001), SCL-90 (−23.00 ± 24.690 vs. −0.96 ± 8.982, *P* < 0.001), and PSQI (−2.95 ± 3.788 vs. 0.61 ± 3.144, *P* = 0.001) compared to the control group. However, despite observed improvement trends in STT-A (−8.81 ± 27.025 vs. 1.70 ± 26.542, *P* = 0.201) and STT-B (−25.90 ± 42.816 vs. −6.83 ± 85.861, *P* = 0.364), the between-group differences were not statistically significant (Figures 4, 5 and Table 4).

**TABLE 4:** Comparison of score changes.

Outcomes	Baduanjin group	Control group	Test statistics	Effect size	95% CI
ITT analysis, Mean (SD)d					
MoCA	3.70 ± 1.941	−0.43 ± 2.428	*t* = 6.373, *P*<0.001*	η^2^ = 0.480	(2.824, 5.437)
MMSE	1.96 ± 1.846	0.04 ± 1.186	*t* = 4.181, *P*<0.001*	η^2^ = 0.284	(0.991, 2.835)
STT-A	−8.04 ± 25.892	1.70 ± 26.542	*t* = −1.260, *P* = 0.214	η^2^ = 0.035	(−25.32, 5.843)
STT-B	−23.65 ± 41.50	−6.83 ± 85.861	*t* = −0.846, *P* = 0.402	η^2^ = 0.016	(−56.901, 23.249)
SCL-90	−21.00 ± 24.456	−0.96 ± 8.982	*t* = −3.690, *P* = 0.001*	η^2^ = 0.236	(−30.992, −9.095)
PSQI	−2.70 ± 3.710	0.61 ± 3.144	*t* = −3.258, *P* = 0.002*	η^2^ = 0.194	(−5.348, −1.261)
PP analysis, Mean (SD)d					
MoCA	4.05 ± 1.627	−0.43 ± 2.428	*t* = 7.122, *P*<0.001*	η^2^ = 0.547	(3.212, 5.752)
MMSE	2.14 ± 1.824	0.04 ± 1.186	*t* = 4.565, *P*<0.001*	η^2^ = 0.332	(1.171, 3.028)
STT-A	−8.81 ± 27.025	1.70 ± 26.542	*t* = −1.300, *P* = 0.201	η^2^ = 0.039	(−26.813, 5.803)
STT-B	−25.90 ± 42.816	−6.83 ± 85.861	*t* = −0.919, *P* = 0.364	η^2^ = 0.020	(−60.099, 22.833)
SCL-90	−23.00 ± 24.69	−0.96 ± 8.982	*t* = −4.005, *P*<0.001*	η^2^ = 0.276	(−33.151, −10.936)
PSQI	−2.95 ± 3.788	0.61 ± 3.144	*t* = −3.404, *P* = 0.001*	η^2^ = 0.216	(−5.672, −1.450)

*Indicates *P*-value is statistically significant.

### 3.5 fNIRS analysis

Owing to excessive motion artifacts, one participant from the control group was excluded from the fNIRS analysis. A 2 × 2 mixed ANOVA was subsequently conducted to compare β-values between the two groups across time points (baseline and 12-week follow-up).

In the Baduanjin group, markedly elevated β-values were identified after 12 weeks of intervention in the following channels and ROIs: CH-26 (*P* = 0.009), CH-38 (*P* = 0.005), CH-47 (*P* = 0.002), CH-50 (*P* = 0.013), CH-55 (*P* = 0.005), CH-58 (*P* = 0.032), CH-63 (*P* = 0.003), as well as in FEF-L (*P* = 0.032) and SSC-R (*P* = 0.007). Conversely, no statistically significant changes were detected in the control group at either time point. Between-group comparisons indicated that the Baduanjin group demonstrated markedly higher β-values in CH-47 (*P* = 0.020) and SSC-R (*P* = 0.013) than those observed in the control group following the 12-week intervention.

Simple effect analysis of channels and ROIs exhibiting significant group × time interactions revealed the following: within the Baduanjin group, activation in CH-47 and SSC-R markedly increased after 12 weeks of training (*P* < 0.01). Furthermore, post-intervention between-group comparisons indicated greater activation in CH-47 and SSC-R in the Baduanjin group compared to the control group (*P* < 0.05).

**FIGURE 6:**
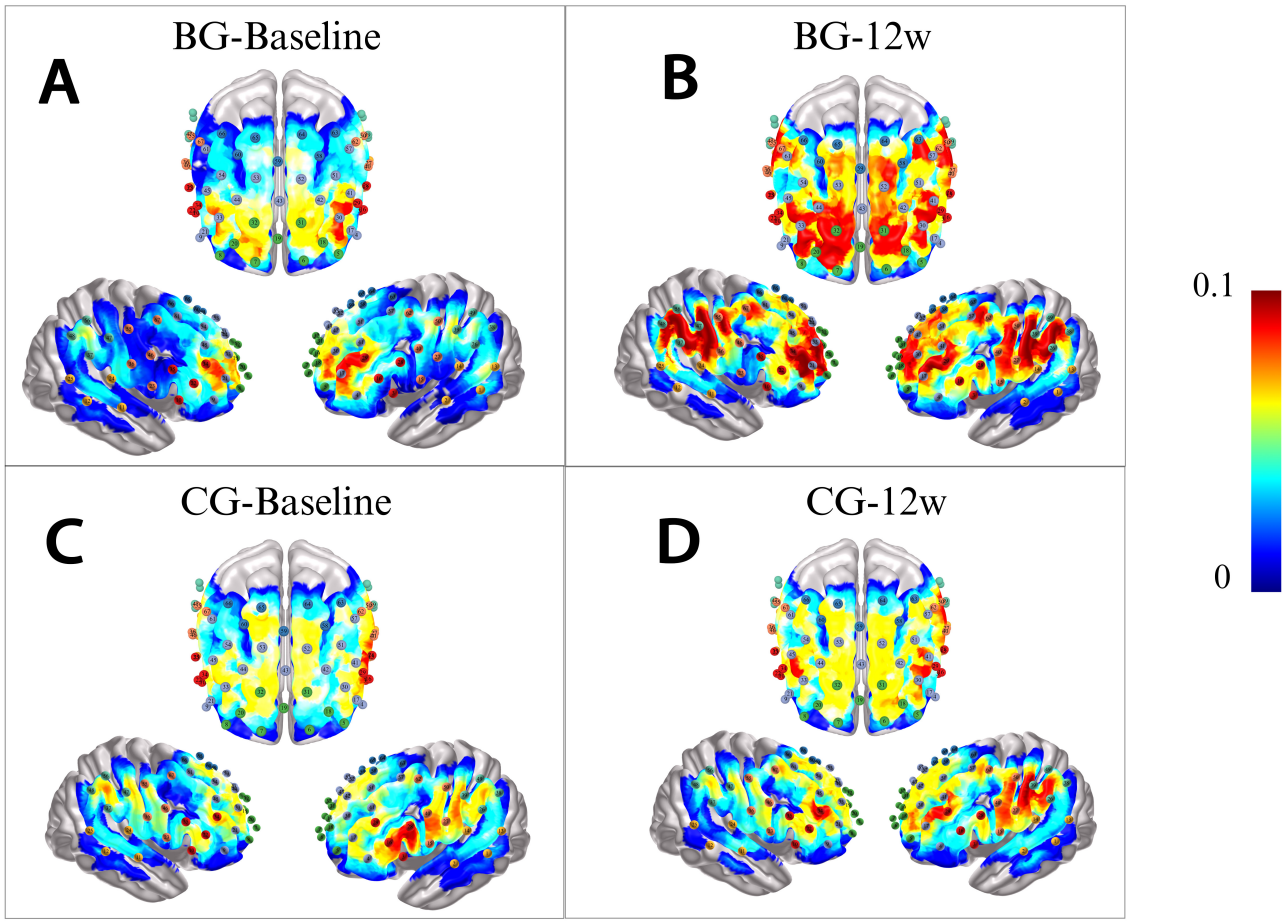
3D activation map. **(A)** Baduanjin Group (BG) baseline 3D activation map. **(B)** Baduanjin Group (BG) after 12w 3D activation map. **(C)** Control Group (CG) baseline 3D activation map. **(D)** Control Group (CG) after 12w 3D activation map.

These findings suggest that Baduanjin training may effectively enhance cognitive function in patients with MCI, with intervention-specific improvements observed in CH-47 and SSC-R beyond changes attributable to natural disease progression. Detailed statistical outcomes are presented in Table 5 and Figure 6.

**TABLE 5:** Comparisons of the β-values during the 1-back tasks in the two groups.

βvalue	Baduanjin group(*n* = 23)			Control group(*n* = 22)			Between-group difference (time × group interaction)	Effect size	95% CI
	Pre	12 wk	Test statistics	Pre	12wk	Test statistics			
CH-26	0.019 ± 0.052	0.061 ± 0.078	0.009*	0.032 ± 0.041	0.045 ± 0.056	0.728	0.727	*d* = 1.143	(−0.027, 0.076)
CH-38	0.016 ± 0.074	0.055 ± 0.047	0.005*	0.036 ± 0.027	0.033 ± 0.036	0.792	0.080	*d* = 1.769	(−0.048, 0.003)
CH-47	0.006 ± 0.117	0.075 ± 0.071	0.002*	0.031 ± 0.042	0.031 ± 0.050	0.983	0.020*	*d* = 2.444	(−0.081, −0.007)
CH-50	0.027 ± 0.072	0.058 ± 0.048	0.013*	0.034 ± 0.039	0.047 ± 0.045	0.218	0.499	*d* = 0.714	(−0.038, 0.019)
CH-55	0.008 ± 0.080	0.062 ± 0.059	0.005*	0.038 ± 0.050	0.033 ± 0.059	0.792	0.097	*d* = 1.667	(−0.065, 0.006)
CH-58	0.014 ± 0.055	0.040 ± 0.024	0.032*	0.028 ± 0.034	0.031 ± 0.043	0.800	0.407	*d* = 0.900	(−0.029, 0.012)
CH-63	0.004 ± 0.083	0.051 ± 0.036	0.003*	0.024 ± 0.027	0.027 ± 0.042	0.825	0.052	*d* = 2.500	(−0.047, 0.000)
FEF-L	0.021 ± 0.055	0.045 ± 0.023	0.032*	0.028 ± 0.028	0.032 ± 0.042	0.765	0.198	*d* = 1.300	(−0.033, 0.007)
SSC-R	0.032 ± 0.056	0.072 ± 0.070	0.007*	0.032 ± 0.040	0.027 ± 0.044	0.697	0.013*	*d* = 2.647	(−0.081, −0.010)

*Indicates *P*-value is statistically significant.

## 4 Discussion

Findings derived from this randomized clinical trial suggest that Baduanjin training markedly enhanced cognitive function among individuals diagnosed with MCI. At the 12-week follow-up, participants in the Baduanjin group demonstrated markedly elevated MoCA and MMSE scores relative to the control group, accompanied by substantially reduced STT-B scores. Furthermore, post-intervention assessments revealed significant declines in both SCL-90 and PSQI scores within the Baduanjin group, with these metrics registering considerably lower than those observed in the control group. Collectively, these outcomes indicate that Baduanjin exercise exerts a beneficial influence on global cognition and certain executive functions in patients with MCI when compared to non-exercise controls, as well as modified cortical activation during performance of the working memory task.

Prior to this investigation, the therapeutic efficacy of Baduanjin in individuals with MCI had not been well established. Although physical exercise has been linked to cognitive improvements in MCI populations, previous studies have yielded inconsistent outcomes regarding both global cognitive performance and specific cognitive subdomains (Law et al., 2020; Song et al., 2018). In this study, fNIRS data indicated that a 12-week Baduanjin intervention markedly enhanced neural activation in Channel 47 (situated within the SSC-R) as well as in the broader SSC-R region, implying that Baduanjin may exert its effects on working memory through neuromodulatory mechanisms involving SSC-R activity.

It is noteworthy that although Baduanjin was expected to enhance activation across the DLPFC, FEF, and SSC, a significant between-group difference was only detected in the SSC-R. This divergence from our initial hypothesis may be attributed to the following reasons. Primarily, Baduanjin may preferentially target the SSC through direct physical-sensory input, whereas its impact on the DLPFC remains more indirect. Thus, the significant SSC-R recruitment highlights Baduanjin’s specific efficacy in bolstering body perception and spatial navigation in the MCI population. Furthermore, the 1-back paradigm might lack the necessary task difficulty to robustly activate the DLPFC, thereby limiting its sensitivity to detect intervention-related improvements in executive control.

This randomized clinical trial confirmed that the Baduanjin intervention facilitated improvements in global cognitive function, aligning with findings from recent investigations (Hong et al., 2024). The cognitive enhancements associated with Baduanjin may be attributed to several underlying mechanisms. As a moderate-intensity aerobic activity, Baduanjin has been found to exert beneficial effects on cognitive performance (Li et al., 2024). Notably, previous research indicates that Baduanjin can modulate functional brain activity via coordinated breathing, particularly influencing the supplementary motor area, premotor cortex, cingulate gyrus, and superior and inferior parietal cortices (Tao et al., 2017). Additionally, Baduanjin training integrates aerobic movements with muscle-strengthening and balance-enhancing components. Its efficacy in promoting muscle strength and improving balance has been demonstrated, particularly in reducing fall risk among elderly individuals (Yuan et al., 2025; Yildirim et al., 2025). Such physical activity may stimulate the secretion of neurochemical mediators such as brain-derived neurotrophic factor and homocysteine, both of which are known to support cognitive function (Liu et al., 2024; Gan et al., 2025). The practice of Baduanjin also engages multiple cognitive domains, including working memory, attentional regulation, and multitasking, while emphasizing the synchronization of movement and breath. This coordination may enhance SSC activation and contribute to neurogenesis and synaptogenesis (Yuan et al., 2023). In the current study, activation of the SSC-R was specifically observed in MCI patients following Baduanjin training. Although the SSC has traditionally been linked to sensorimotor processing, recent evidence suggests its involvement in working memory tasks, such as those requiring tactile and spatial information (Guidali et al., 2020). The SSC-R, in particular, appears to serve a primary function in spatial working memory and the allocation of attentional resources (Esmaeili and Diamond, 2019). Three specific movements in Baduanjin—Holding the Sky with Two Hands to Regulate the Triple Burner, Drawing the Bow to Shoot the Eagle, and Swaying the Head and Shaking the Tail to Relieve Heart Fire—require continuous spatial orientation and proprioceptive processing, which may therefore preferentially activate the SSC-R.

In the ITT analysis, although the between-group difference in STT-A scores between the Baduanjin group and the control group was statistically significant after intervention, neither the intragroup comparison before and after treatment nor the between-group comparison of the changes in STT-A scores showed statistical significance. For STT-B, the between-group difference in change scores was not statistically significant; however, the between-group difference after treatment was significant, the intragroup improvement in the Baduanjin group was significant, and the PP analysis consistently yielded positive results. The lack of significance in the change scores may be related to the dilution of intervention effects caused by the inclusion of dropouts in the ITT population. Collectively, these findings demonstrated that Baduanjin training led to significant improvements in global cognitive function among patients with MCI, with notable enhancements in executive function, as reflected by elevated MoCA and MMSE scores and shortened completion times on the STT-B. The intervention was also associated with improvements in emotional state and sleep quality, as evidenced by reductions in SCL-90 and PSQI scores. The discrepancy between STT-A and STT-B results suggests that Baduanjin may exert greater influence on higher-order cognitive processes, specifically executive control, while its impact on basic cognitive tasks remains relatively limited. Additionally, the relaxation elements embedded in Baduanjin practice may contribute to reductions in anxiety and depression, potentially through the modulation of cortisol levels and other stress-related biological pathways implicated in cognitive deterioration.

Previous investigations have reported comparable findings indicating that aerobic exercise may enhance cognitive function through the modulation of specific brain regions. For instance, Tai Chi has been shown to effectively increase gray matter volume in the right frontal gyrus, precentral gyrus, and occipital gyrus among individuals with MCI (Zheng et al., 2021). Nonetheless, limited attention has been directed toward the SSC-R. To our knowledge, the present study is to identify the distinct effects of Baduanjin exercise on SSC-R, thereby contributing to a broader understanding of the SSC’s involvement in cognitive intervention.

This study possesses several noteworthy strengths. In contrast to prior investigations, both ITT and PP analyses were performed, providing comprehensive effect estimates across all outcome measures. Furthermore, the exercise intervention was associated with a low attrition rate, enhancing the robustness of the findings. Several limitations of the present study warrant acknowledgment. First, the sample size calculation was based on an effect size (d) derived from a pilot study, which may be relatively large and thus optimistic. Second, the study employed a multifaceted analytical framework involving multiple behavioral outcomes and a series of fNIRS channel and ROI analyses. Given that the fNIRS correlation analyses were not corrected for multiple comparisons, these findings should be interpreted as exploratory and preliminary. Furthermore, the control group design was relatively weak, which precludes the differentiation of Baduanjin’s specific therapeutic components from non-specific factors, such as social interaction or instructor supervision. Consequently, the current results necessitate validation through larger-scale, well-powered randomized controlled trials. Future research should implement more rigorous control conditions to isolate the specific effects of Baduanjin and strengthen the causal interpretation of the intervention. Nevertheless, despite these constraints, this study provides valuable preliminary evidence regarding the impact of Baduanjin on cognitive function and task-related cortical activation in patients with MCI.

## 5 Conclusion

Baduanjin training can improve cognitive function in MCI patients, particularly working memory and executive function, which may be associated with increased activation in the SSC-R.

## Data Availability

The raw data supporting the conclusions of this article will be made available by the authors, without undue reservation.
